# Effects of drop net and helicopter net-gun capture on movement, space use, and survival of white-tailed deer

**DOI:** 10.1371/journal.pone.0340491

**Published:** 2026-01-06

**Authors:** Dylan G. Stewart, Jared T. Beaver, M. Lucas Cooksey, Chad Grantham, Brian L. Pierce, Roel R. Lopez, Stephen L. Webb

**Affiliations:** 1 Department of Rangeland, Wildlife, and Fisheries Management, Texas A&M University, College Station, Texas, United States of America; 2 Department of Animal and Range Sciences, Montana State University, Bozeman, Montana, United States of America; 3 Texas A&M Natural Resources Institute, San Antonio, Texas, United States of America; 4 Natural Resources Conservation Service, United States Department of Agriculture, Waxahachie, Texas, United States of America; 5 Texas A&M Natural Resources Institute, College Station, Texas, United States of America; Colorado State University College of Veterinary Medicine and Biomedical Sciences, UNITED STATES OF AMERICA

## Abstract

Wildlife professionals strive to minimize the impact of capture and handling on animal welfare and behavior. Drop net and helicopter net-gun methods are commonly used to capture large mammals; however, comparative evaluations of mortality and post-capture behavioral disruption are needed. Therefore, we conducted a study to compare the effects of drop net, single helicopter, and tandem helicopter capture methods on movement, space use, and survival of white-tailed deer (*Odocoileus virginianus*) for 30 days following capture. We fitted 149 deer (68 F; 81 M) with GPS collars, which collected or were resampled to 1 location every 30 minutes from 2011 to 2015. We estimated daily range size and overlap using 99% range isopleths, calculated daily distance traveled and displacement, and fitted Cox proportional hazards and Kaplan-Meier models to assess survival and estimate survival rates. Survival was similar among capture methods (drop net, *S* = 0.90; single helicopter, *S* = 0.94; tandem helicopters, *S* = 0.94) and was not affected by capture method, capture season, or sex. Deer movement and space use were largely unaffected by capture, with movement returning to long-term means within 1–2 days of capture, but as long as 9 days following helicopter captures. Drop nets had the least effect, likely because capture sites were within or near previously used areas, whereas deer captured by helicopter were transferred 0.5 to 3.1 km. Our findings indicate that these capture methods are effective and have minimal impacts on deer movement, space use, and survival. Helicopter capture methods can be more economical and efficient, but drop nets may pose lower risks to human safety and may be necessary in areas where helicopter access is limited. Our findings provide guidance for wildlife professionals to select capture methods that minimize behavioral modifications while meeting project objectives.

## Introduction

Effective management of free-ranging wildlife populations often relies on key information gathered through capturing, handling, marking, and tracking animals [1–3]. In addition to collecting biological and morphological data, researchers often fit captured animals with global positioning system (GPS) devices, which are a cost-effective, passive tool used for collecting large amounts of spatial and temporal data [4–6]. Location data collected from tracking devices can be used to construct home range estimates, detect mortality events, estimate parturition dates, quantify intra- and interspecific contacts, determine habitat selection, and relocate disease-infected animals [2,3,7–10]. Although capturing and tracking animals provides valuable data, it can also lead to unintended consequences, including direct or indirect mortality, injury, separation from maternal or social groups, and disruption of normal behavior [11–13].

Researchers must balance project objectives with economic and logistical limitations, necessitating capture methods that are not only safe for both animals and humans but also cost-effective and efficient [14,15]. Drop net and helicopter net-gun capture techniques are widely regarded as safe capture techniques for many large terrestrial mammals (e.g., deer [*Odocoileus* spp.], ≤ 7% mortality; [16–18]). However, there is an inherent risk to human safety when using helicopters, particularly when operating tandem helicopters within the same area [19]. Both capture methods allow for selectivity based on sex and age class [18,20,21]; however, drop net captures are more passive, relying on animal presence, and tend to favor younger age classes [15,22]. This is in comparison to helicopter net-gun captures where animals are actively located and captured. Both methods can be conducted without the use of chemical immobilization [18,20], which is encouraged when possible [22].

The helicopter net-gun technique has been used to capture large mammals in a range of environments, including shrub-dominated landscapes [18], but is most successful in open landscapes [23–26]. Landscapes with dense overstory cover can make it more challenging to locate and capture animals and may pose increased safety risks to helicopter crews [14,25–27]. In contrast, drop nets are effective across most landscapes [14,20,28], but can be more time intensive and less cost-effective than the helicopter net-gun technique [14,15], depending on factors related to the density of the target species, capture effort, and landscape composition.

In addition to human and animal safety, researchers strive to minimize the short-term effects of capture on animal health and behavior to ensure animal welfare [22,29]. Research suggests that animals are affected by the capture and handling process; therefore, these anthropogenic disturbances should be accounted for during data analysis to avoid potential biases resulting from capture-related changes in animal behavior [30–34]. Animals have differing physiological, biochemical, and hematological responses to capture, which influence the magnitude of the capture effects and recovery period of animals following capture [29]. In several species, the helicopter net-gun capture technique affected movement and space use of resident animals for up to 10 days following capture [24,35–37]. Published data on space use and movement following drop net captures is scarce. However, it is generally found or assumed that animals will be affected for up to 10 days post capture, but certain behavioral effects may extend multiple weeks [30,32,33].

Although drop net and helicopter net-gun techniques are commonly used to capture large mammals, direct comparisons between methods are rare [36–38] and little information exists on the differences in magnitude of effect and recovery duration as it relates to animal movement and space use. Therefore, we designed a study, building on previous findings regarding effectiveness and cost [15], to compare the impacts of drop nets, single helicopter, and tandem helicopter capture methods on space use, movement, and survival of white-tailed deer (*Odocoileus virginianus*) with the goal of informing wildlife professionals about the trade-offs associated with each method. We predicted that deer captured using drop nets would return to normal behavior more quickly and have higher survival rates compared to those captured by helicopter net-gunning techniques. This prediction is predicated on the ability to deploy drop nets at baited sites within an animal’s home range, likely reducing the need to search for refuge after capture and minimizing the associated stress. In contrast, deer captured by helicopter may be moved outside their established ranges, potentially prolonging the period of elevated stress [24]. Additionally, net-guns fired from helicopters use weighted projectiles, which may occasionally cause incidental injuries (e.g., broken legs) that could lead to death [18,23,39].

## Materials and methods

### Study area

**Fig 1:**
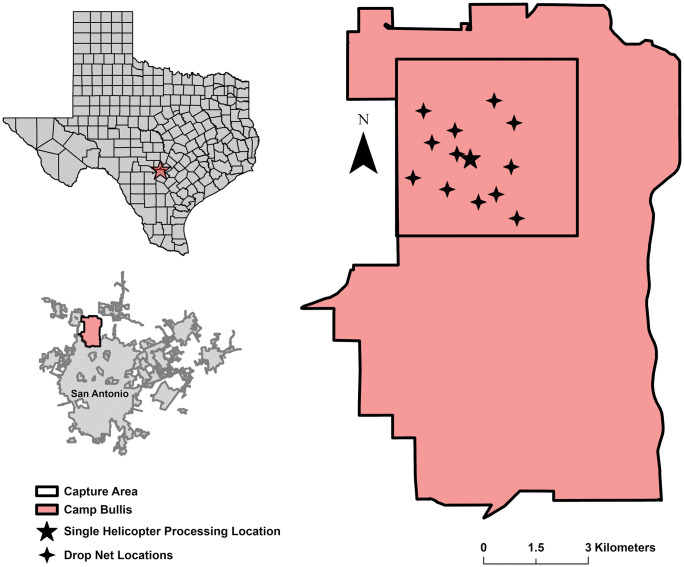
Study site. Drop net capture locations (*n* = 12; four-point stars), central helicopter processing location (five-point star) for single helicopter captures, and the capture area (black outline) for white-tailed deer (*Odocoileus virginianus*) research on Joint Base San Antonio-Camp Bullis, Texas, USA, 2011–2015. Map was developed by **D. G.** Stewart using ArcGIS Pro v3.5.3 (Esri, Redlands, CA, USA).

We conducted our research at Joint Base San Antonio-Camp Bullis (hereafter, Camp Bullis), a 11,286-ha U.S. Army training facility located north of San Antonio within the Edwards Plateau, Blackland Prairies, and South Texas Plains ecoregions, Bexar County, Texas, USA ([15,40]; Fig 1). We limited deer capture efforts to a 2,500-ha portion of Camp Bullis because it was representative of routine military activities (e.g., troop training exercises, live range practice) that aligned with research objectives while maintaining the safety of military personnel ([15], Fig 1). Camp Bullis was enclosed with a ~ 2.4-m tall high fence constructed from 9-gauge galvanized steel wire chain link mesh and 3 strands of barbed wire at the top of the fence (and projecting outward from the property) to provide security [41]. The fence design also limited ingress and egress of deer [42].

The two dominant vegetation cover types on Camp Bullis were scrub evergreen and upland deciduous forest [15,43,44]. Scrub evergreen forests were dominated by Ashe juniper (*Juniperus ashei*), plateau live oak (*Quercus virginiana*), and Texas persimmon (*Diospyros texana*; [15,44]), whereas upland deciduous forests were an interspersion of Spanish oak (*Quercus texana*), Lacey oak (*Quercus glaucoides*), Ashe juniper, and Texas persimmon [15,44]. The elevation of Camp Bullis ranged from 320 to 440 m above sea level [45]. Soils consisted of Brackett gravelly clay loam (39%), Krum clay (15%), and Eck-rant cobbly clay (25%; [46]). Annual temperature and precipitation ranged from 11 to 30°C (July) and from 36 to 89 cm, respectively [15].

### Capture and handling

#### Drop net captures.

We captured deer using drop nets in August, September, and November 2011 and January, February, March, and June 2012. We deployed 6 drop nets at 12 baited sites, alternating net placement based on deer activity detected by remote cameras [15]. We captured deer using 20 × 20-m knotless nylon drop nets, baited with shelled corn, and released manually by a researcher stationed nearby in a hunting blind [15,47]. Drop net sites were selected to provide uniform coverage across the study site, and timing of trapping at each site was based on deer use (Fig 1; [15]). Trapping occurred four days per week from August to November and continuously from February to April [15]. Captured deer were processed and released at the capture site [15].

#### Single helicopter captures.

We captured deer using a net-gun deployed from a single helicopter in July 2012 and February 2013. A pilot and net-gunner used a Robinson R22 helicopter (Robinson Helicopter Company, Torrance, CA, USA) to pursue deer into open areas, flying 4–6 m above the animals [15]. Each pursuit lasted <10 minutes to reduce the risk of overheating [15]. Deer were captured using a 4-barreled net gun (Holt Helicopters Inc., Uvalde, TX, USA; [48,49]), hobbled by the gunner, and transported via cable to a central processing station (0.5–3.1 km from capture site; [15]).

#### Tandem helicopter captures.

We captured deer using a net-gun as part of a tandem helicopter team during July 2013, February, April, and July 2014, and October 2015. The tandem helicopter method incorporated the use of two helicopters (Smith Helicopters Inc., Cotulla, TX, USA) operating in coordination, where one netted and hobbled deer, while the second assisted with locating, flushing, and transporting animals [15,49]. Consistent with single helicopter captures, we limited helicopter pursuits to <10 minutes to reduce the potential for overheating [15]. Deer were not transported to a central station; instead, two mobile processing units followed the helicopters along main roads, ensuring processing occurred within 0.5 km of the capture site. Deer were released from the processing sites [15].

#### Deer handling.

We blindfolded, manually restrained, affixed uniquely numbered ear tags, aged using the tooth replacement and wear method [50], sexed, scored body condition, recorded the processing time, and fitted each deer with a Sirtrack GPS collar (Model G2C 191; Sirtrack, Havelock North, New Zealand; [15]). To further minimize the risk of overheating, we captured deer during daylight hours from sunrise to 1100 CDT/CST and reduced processing time by not collecting biological samples that could prolong handling (e.g., blood draws). We classified capture events into one of 4 categories based on meteorological seasons [51]: spring (March–May), summer (June–August), autumn (September–November), and winter (December–February). All animal capture and handling protocols were approved by the Texas A&M University Institutional Animal Care and Use Committee (AUP#: 2011−154).

### GPS data collection and management

We programmed GPS collars to collect 1 location every 15-minutes (96 fixes/day) or 30-minutes (48 fixes/day). We downloaded location data following collar retrieval, resampled location data collected at 15-minute intervals to 30-minute fix intervals (48 locations/day) for consistency, converted timestamps from Coordinate Universal Time+0 (UTC + 0) to local mean time (LMT), recorded the time zone (CDT [UTC-5], CST [UTC-6]), and calculated the Euclidean distance, velocity, net displacement, turning angle, heading, and time between sequential locations.

We used methods described by Stewart et al. [42] to confirm or estimate when each collar detached or a mortality event occurred (i.e., end date) and to identify and censor erroneous GPS fixes while minimizing data loss. First, we used a function from the amt package [52] in R statistical software [53] to predict when the event occurred. We identified the first location within the initial stationary cluster detected by the function as the collar drop or deer mortality date. Next, we used a function from the ctmm package [54,55] to identify locations that were >32 km from the median longitude and latitude of locations for each collar deployment. We also identified locations ≥2 km from an established location cluster within a 15–30-minute period. We then censored these erroneous and unrealistic locations to avoid bias associated with inaccurate positions.

To assess deer survival following capture, we subsampled deer location data to include the day of capture and the subsequent 30 days, resulting in a 31-day study period. We focused on this interval because mortalities within the first month may be capture-related (e.g., capture myopathy) as reported in previous studies [39,56–58]. We evaluated data starting the day of capture because capture myopathy can occur immediately following release [39,56–58]. We monitored collared deer weekly to determine cause of mortality, when possible, which was difficult given the weekly monitoring schedule. All mortalities occurring within the first 31 days were included in survival analyses [15,39,56,59], while individuals surviving beyond 31 days were treated as survivors for the purposes of this analysis [59].

We subsampled location data to the first 30 days following the day of capture to evaluate the effect of day since capture, sex (female, male), capture method (drop net, single helicopter, tandem helicopters), and capture season (spring, summer, autumn, winter) on deer movement and space use. We censored location data from the day of capture because capture times varied, so the amount of data on the day of capture varied and could impact movement and space use metrics [36]. We also censored collars from movement and space use analyses that collected <30 days of continuous location data to avoid bias from data loss and avoid the confounded effect of reduced movement among deer suffering from capture myopathy [57]. We conducted space use and movement analyses independent of survival analyses, so collars could be included in survival analyses but censored from space use and movement analyses. We selected 30 and 31-day study periods because similar studies have reported that the effect of capture on large mammals typically dissipates within the first 30 days following release [24,34,36,60,61].

### Data analysis

We evaluated the effect of capture method, sex, and capture season on survival and estimated survival rates to complement and expand upon the summary findings of Beaver et al. [15] using additional data and covariates. Specifically, we used functions from the survival package [62,63] to fit a semi-parametric Cox proportional hazards (CPH) model with time to mortality as the response variable, a categorical explanatory variable for capture method with 3 levels (drop net, single helicopter, tandem helicopter), a categorical explanatory variable for sex with 2 levels (female, male), and a categorical explanatory variable for capture season with 2 levels (warm, cool). We combined and reclassified spring, autumn, and winter seasons into a single category (cool) because we expected extreme summer (warm) temperatures to have the greatest effect on survival, and because there were too few mortalities in spring (*n* = 2) and autumn (*n* = 1) to include as separate levels. We did not include interaction terms in the CPH model because the limited number of mortality events did not occur across most levels of the 3 factors, which led to convergence issues. We evaluated the significance of fixed effects on time to mortality using Type III analysis of deviance (Wald χ² tests) implemented using a function from the car package [64]. This approach tests each effect after accounting for all other terms in the model, providing χ² statistics, degrees of freedom, and associated *P*-values. We reported the concordance index (C-index) to assess model discrimination, which quantifies the agreement between observed outcomes and model predictions [62,63]. A C-index of 0.5 indicates no predictive ability (random chance) whereas a value of 1 indicates perfect prediction [62,63]. We evaluated the proportional hazards assumption using scaled Schoenfeld residuals [62,63].

To visualize unadjusted survival patterns, we used functions from the survival package to generate 4 non-parametric Kaplan–Meier (KM) models, each with time to mortality as the response variable and either capture method, sex, capture season, or the null model as the explanatory variable. These KM models provided estimates of survival probability (*S*) used to describe uncorrected survival across groups.

We leveraged subhourly location data to evaluate the effect of day since capture, sex, capture method, and capture season on daily distance traveled (m), mean daily displacement (m), daily range size (ha), and daily range overlap (0–1) of white-tailed deer over a 30-day period following capture. We chose to evaluate range size and overlap because they are the most basic and most widely utilized indices of space use and are influenced by the spatial arrangement and availability of resources [65,66]. Similarly, we analyzed distance traveled and displacement as indicators of deer movement because these metrics are widely used in ecological research, are directly comparable across studies, and are influenced by the spatial distribution and availability of resources [66–69]. We used a function from the adehabitatHR package [70] to create 99% daily kernel range isopleths for deer using the reference bandwidth (href) and a 100-m grid and functions from the sf [71,72] and rgeos [73] packages to estimate range size (ha) and proportion of overlap (0–1) between daily ranges. We calculated estimates of range overlap for 29 of the 30 days in the study period, as estimating 29 daily range overlaps requires 30 consecutive daily ranges. We quantified daily distance traveled (m/day) by adding sequential step lengths (i.e., Euclidean distance between two GPS locations, termed a step) and calculated mean daily displacement (m/day) by averaging the Euclidean distance from each GPS location to the first GPS location of the day following release.

Previous studies have evaluated the effect of capture on movement and space use of large mammals by comparing metrics after capture to some predetermined time frame that represents “normal activity” [34,38,60,74]. We chose a similar approach; however, instead of using the literature to predict when normal activity likely occurred [34], we created a long-term mean, which represented normal activity for each individual deer by averaging the movement and space use metrics for each deer across the 30-day period [74]. We included 30 days of data, which extends well beyond the period most animals are affected by capture [34], to minimize bias from altered post-capture behavior that could otherwise influence estimates of the long-term mean [74]. To account for variation within and among deer, we treated individual deer as the sampling unit when calculating 30-day means and for calculating relative effect sizes for statistical analysis.

We hypothesized that day since capture, capture method, sex, and capture season would be important predictors of deer movement and space use. To evaluate the effects, we used a function from the glmmTMB package [75] to create generalized linear mixed effects models (GLMMs); we developed one model for each metric of movement (distance traveled, displacement) and space use (range size, overlap). We developed 4 models (*n* = 4), each with a continuous response variable for each movement and space use metric, a categorical explanatory variable for day since capture (reference = long-term mean, days 1–30), a categorical explanatory variable for capture method with 3 levels (drop net, single helicopter, tandem helicopter), a categorical explanatory variable for sex with 2 levels (female, male), and a categorical explanatory variable for capture season with 4 levels (spring, summer, autumn, winter), which was included as a blocking factor to account for seasonal differences. We specified interactions among day since capture, capture method, and sex. Prior to analysis, we used a function from the bestNormalize package [76,77] to identify and apply the transformation that best normalized each response variable. We specified a Gaussian distribution and an identity link function for all models, and we included a random intercept term for the unique deer collaring event to account for individual variation [78] and reduce autocorrelation within individuals [79].

We used functions in the DHARMa package [80] to create QQ and residual plots, which we used to evaluate model diagnostics and confirm that a Gaussian distribution with an identity link function appropriately fit the data. We used a similar approach as described for evaluating terms in the CPH model, implementing a Type III analysis of deviance (Wald χ² tests) to assess the significance of each effect while accounting for all other terms in the model. We used a function from the ggeffects package [81] to generate marginal (population-level) model-based predictions of movement and space use. Predictions were bias-corrected and averaged across the levels of other factors in the model. Predicted values were backtransformed for ease of interpretation and comparison.

We used functions from the emmeans package [82] to compute estimated marginal means (EMMs) and perform pairwise contrasts, comparing each day to the long-term mean (reference) in a treatment-vs-control framework. Comparisons were conducted separately within each level of the categorical predictors, and days that differed significantly from the long-term mean (α = 0.05) were considered indicative of altered movement or space use. We considered an estimate to represent altered movement or space use if it significantly differed from the long-term mean and occurred immediately following capture, or if it was a single non-significant day embedded within a sequence of estimates that significantly differed from the long-term mean. The latter approach accounts for daily variation while capturing the overall trend in altered movement or space use. Days identified as significantly different from the long-term mean, but appearing randomly throughout the time series, were considered artifacts of daily or seasonal variation rather than effects of capture. When significant interactions involving capture method were detected, we computed EMMs and pairwise contrasts to compare movement and space use indices among capture methods. If sex was included in a significant interaction, comparisons were conducted separately within sex. For visualization, we focused on the highest-order interaction in the model because it represents the combined effect of all main factors. Lower-order main effects and interactions were summarized in the Results section. Last, if season of capture significantly impacted movement or space use, we calculated EMMs for capture period and performed pairwise comparisons among levels using Tukey-adjusted contrasts.

## Results

### Deer sampled

We captured 202 individual deer, including 18 deer that were captured more than once (16 recaptured once, 2 recaptured twice), totaling 222 unique capture events. We censored 32 capture events because capture data were missing or the collar was damaged, malfunctioned, or unrecovered, resulting in loss of data.

We analyzed 190 unique capture events of 174 deer (82 F; 92 M) to estimate 31-day survival rates. Of the 190 unique captures, 31 deer (9 F; 22 M) were captured by drop nets, 65 (30 F; 35 M) by single helicopters, and 94 (51 F; 43 M) by tandem helicopters. Three deer (1 F; 2 M) were direct capture-related mortalities (1 drop net, 1 single helicopter, 1 tandem helicopter) and were included in survival analyses but were censored from movement and space use analyses. Of those included in survival analyses, 6 females and 6 males were recaptured once, and 1 female and 1 male were recaptured twice.

The mean age of deer at capture was 3.1 years (± 0.1 SE, range = 0.5–6.5 years; [42]). The mean processing time, not including chase time, of captured deer was 22.2 (*n* = 13, SE = 1.9, range = 11–36), 6.2 (*n* = 64, SE = 0.3, range = 3–15), and 5.1 minutes (*n* = 92, SE = 0.2, range = 2–10) for drop net, single helicopter, and tandem helicopter captures, respectively [42].

### Collar efficacy

We collected 230,808 GPS locations from collars fitted on 149 individual deer (68 F; 81 M), representing 162 unique capture events, that collected location data for the full 30-day study period. Of the 190 unique capture events, we censored 25 collars from space use and movement analyses due to mortality within the 30-day study period (*n* = 10) and because some collars collected fewer than 30 days of location data (*n* = 15). Three deer died as the result of capture-related mortality and were not fit with a collar, which summed to 28 censored capture events. Collars collected an average of 1,424 fixes ± 3.9 (SE) over the 30-day study period, respectively. The average fix success rate after data censoring and resampling was 99% ± 0.3 (SE).

### Survival

We documented 13 mortalities (6.8% of 190 capture events) within the first 31 days (day of capture and subsequent 30 days) following capture. Three (1.6%) were the direct result of capture by drop net (*n* = 1), single helicopter (*n* = 1), and tandem helicopters (*n* = 1) capture methods. The remaining 10 mortalities (5.3%) occurred following captures conducted with drop nets (*n* = 2), single helicopters (*n* = 3), and tandem helicopters (*n* = 5). We documented 2 mortalities in spring, 1 in autumn, and 5 in winter, totaling 8 mortalities during the cool season, and 5 mortalities in summer, reclassified as the warm season. The average time to mortality was 7.1 days (*n* = 10, SE = 1.7, range = 1–19) for deer that died following release.

**Fig 2:**
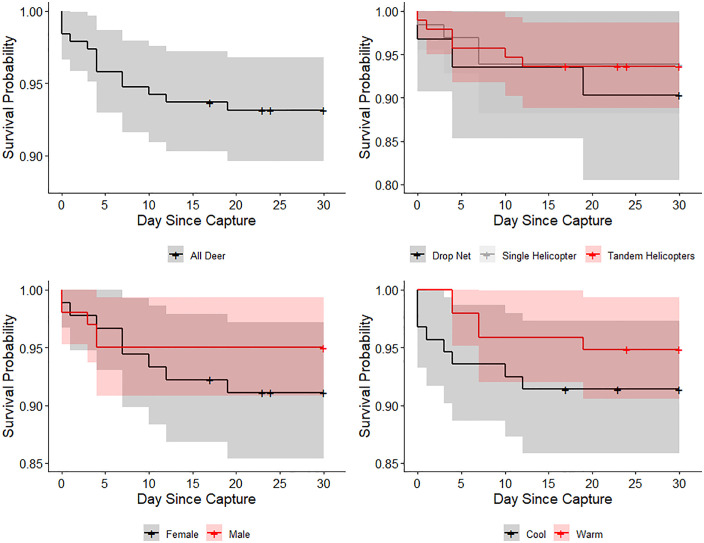
Factors affecting white-tailed deer survival. The effects of (a) day since capture (day 0–30), (b) capture method (drop net, single helicopter, and tandem helicopter), (c) sex (female, male), and (d) capture season (cool [spring, autumn, winter], warm [summer]) on Kaplan-Meier survival estimates (with 95% CI) of white-tailed deer (*Odocoileus virginianus*) on Joint Base San Antonio-Camp Bullis, Texas, USA, 2011–2015.

The concordance index (C-index) of the CPH model was 0.62 ± 0.08 SE, indicating moderate predictive ability. The proportional hazards assumption was not violated for the overall model (Global test: χ² = 5.92, df = 4, *P* = 0.21). Survival was not affected by capture method (χ² = 0.42, df = 2, *P* = 0.81), capture season (χ² = 0.80, df = 1, *P* = 0.37), or sex (χ² = 1.44, df = 1, *P* = 0.23). The uncorrected 31-day survival (*S*) across all deer was 0.93 ± 0.02 SE (Fig 2a). Survival estimates were 0.91 ± 0.03 SE for females and 0.95 ± 0.02 SE for males (Fig 2c). By capture method, survival was 0.90 ± 0.05 SE (drop net), 0.94 ± 0.03 SE (single helicopter), and 0.94 ± 0.03 SE (tandem helicopter; Fig 2b). By season, survival was 0.91 ± 0.03 SE during the cool season and 0.95 ± 0.02 SE during the warm season (Fig 2d).

### Movement and space use

**Fig 3:**
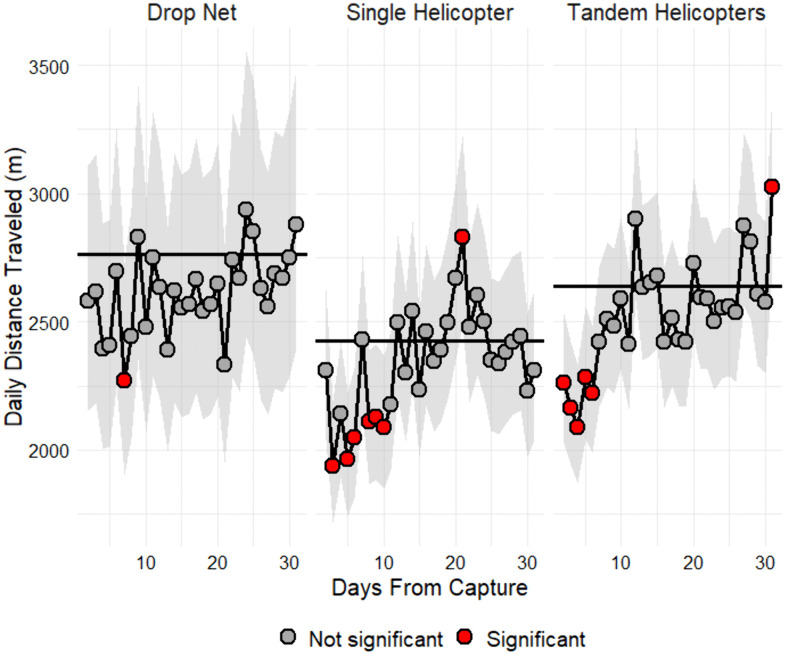
Effect of capture method and day since capture on daily distance traveled of white-tailed deer. Effect of capture method (drop net, single helicopter, tandem helicopters) and day since capture (long-term mean, days 0–30) on daily distance traveled (m/d) of white-tailed deer (*Odocoileus virginianus*) on Joint Base San Antonio-Camp Bullis, Texas, USA, 2011–2015. Daily estimates that significantly (*P* ≤ 0.05) deviated from the 30-day mean (black solid line) are represented by red points.

The Yeo-Johnson transformation (yeojohnson) provided the best normalization for daily distance traveled (m/d). Daily distance traveled was affected by capture season (χ² = 15.98, df = 3, *P* = 0.001) and the interaction between day since capture and capture method (χ² = 96.93, df = 60, *P* = 0.002) but not by other main effects or interactions (*P* > 0.05). Daily distance traveled did not differ between capture methods (*P* > 0.05). Distance traveled by deer captured by drop nets did not differ significantly from the 30-day long-term mean (Fig 3). In contrast, distance traveled decreased significantly for 9 days following single helicopter capture and for 5 days following tandem helicopter capture (Fig 3). Predicted daily distance traveled (m) was greatest in autumn (3,660 m, 95% CI [2,959, 4,562]), least in spring (2,257 m, 95% CI [1,601, 3,246]), and similar in summer (2,765 m, 95% CI [2,302, 3,339]) and winter (2,746 m, 95% CI [2,296, 3,301]). Daily distance traveled (transformed) was significantly less during summer compared to autumn (estimate = –0.72, SE = 0.21, t = –3.49, *P* = 0.002) and greater during autumn compared to spring (estimate = 1.26, SE = 0.48, t = 2.65, *P* = 0.04) and winter (estimate = 0.74, SE = 0.21, t = 3.50, *P* = 0.003) but did not differ between other seasons (*P* > 0.05). The average 30-day long-term model-predicted marginal mean was 2,765 m (95% CI [2,302, 3,339]), 2,426 m (95% CI [2,140, 2,758]), and 2,641 m (95% CI [2,358, 2,964]) for deer captured by drop net, single helicopter, and tandem helicopters, respectively (Fig 3).

**Fig 4:**
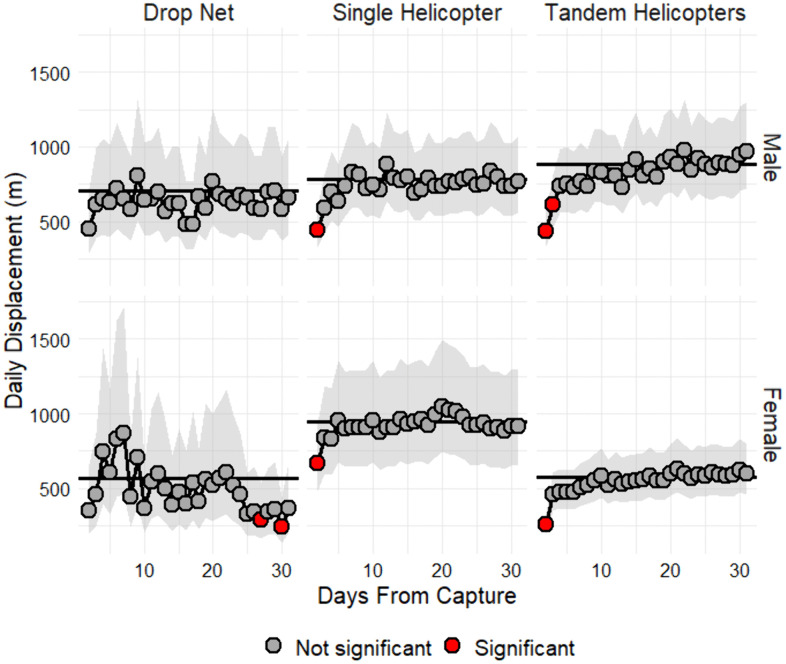
Effect of capture method, day since capture, and sex on daily displacement of white-tailed deer. Effect of capture method (drop net, single helicopter, tandem helicopters), day since capture (long-term mean, days 0–30), and sex (female, male) on daily displacement (m/d) of white-tailed deer (*Odocoileus virginianus*) on Joint Base San Antonio-Camp Bullis, Texas, USA, 2011–2015. Daily estimates that significantly (*P* ≤ 0.05) deviated from the 30-day mean (black solid line) are represented by red points.

The Ordered Quantile transformation (orderNorm) best normalized daily displacement (m/d). Daily displacement was affected by day since capture (χ² = 56.43, df = 30, *P* = 0.002), capture season (χ² = 27.81, df = 3, *P* < 0.001), the interaction between day since capture and capture method (χ² = 87.56, df = 60, *P* = 0.01), the interaction day since capture and sex (χ² = 101.17, df = 30, *P* < 0.001), and the interaction among day since capture, capture method, and sex (χ² = 116.08, df = 60, *P* < 0.001) but not by other main effects or interactions (*P* > 0.05). Daily displacement of male deer did not differ among capture methods (*P* > 0.05). Daily displacement (transformed) of female deer was greater when captured by a single helicopter compared to tandem helicopters (estimate = 0.56, SE = 0.20, t = 2.77, *P* = 0.02) but did not differ between other capture method comparisons (*P* > 0.05). Daily displacement of female and male deer captured by drop nets did not differ significantly from long-term means (Fig 4). Whereas daily displacement of female and male deer captured by a single helicopter and female deer captured by tandem helicopters was significantly reduced for one day following capture; daily displacement of male deer captured by tandem helicopters was significantly reduced for 2 days following capture (Fig 4). Predicted daily displacement (m) was greatest in autumn (1,337 m, 95% CI [752, 2,455]) and winter (1,397 m, 95% CI [871, 2,289]) and least in spring (835 m, 95% CI [280, 2,810]) and summer (706 m, 95% CI [445, 1,144]). Daily displacement (transformed) was significantly less during summer compared to winter (estimate = –0.70, SE = 0.14, t = –5.08, *P* < 0.001) but did not differ between other seasons (*P* > 0.05). The average long-term model-predicted marginal mean was similar for male deer captured by drop net (706 m, 95% CI [445, 1,144]), single helicopter (783 m, 95% CI [567, 1,092]), and tandem helicopters (882 m, 95% CI [662, 1,185]) (Fig 4). The average long-term model-predicted marginal mean was similar for female deer captured by drop net (564 m, 95% CI [306, 1,080]) and tandem helicopters (576 m, 95% CI [437, 765]) but considerably greater for those captured by a single helicopter (948 m, 95% CI [674, 1,348]) (Fig 4).

**Fig 5:**
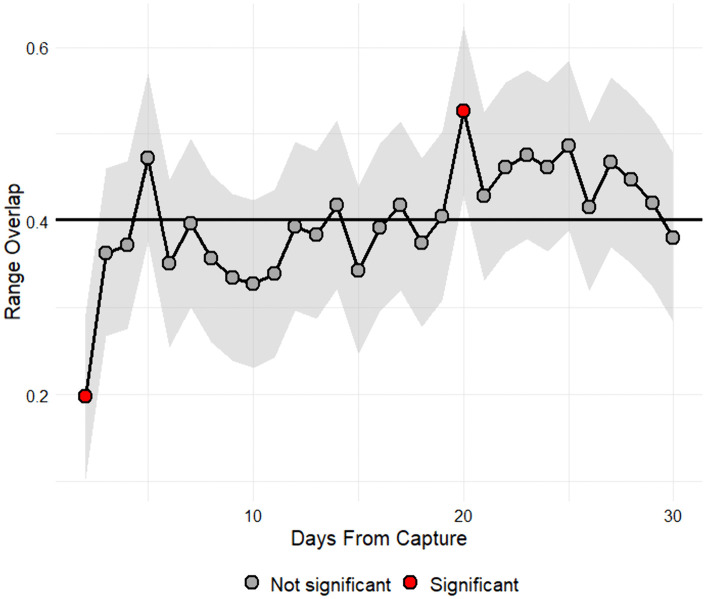
Effect of day since capture on daily range overlap of white-tailed deer. Effect of day since capture (long-term mean, days 0–30) on daily range overlap (0-1) of white-tailed deer (*Odocoileus virginianus*) on Joint Base San Antonio-Camp Bullis, Texas, USA, 2011–2015. Daily estimates that significantly (*P* ≤ 0.05) deviated from the 30-day mean (black solid line) are represented by red points.

The Ordered Quantile transformation best normalized range size (ha) and overlap (0–1). Range size was affected by capture season (χ² = 8.47, df = 3, *P* = 0.04) but not by other main effects or interactions (*P* > 0.05). Range overlap was affected by main effects for day since capture (χ² = 50.09, df = 29, *P* < 0.001) and capture season (χ² = 11.76, df = 3, *P* = 0.008) but not by other main effects or interactions (*P* > 0.05). Predicted range size (ha) was 177 ha (95% CI [75, 428]) in spring, 281 ha (95% CI [169, 472]) in summer, 387 ha (95% CI [222, 682]) in autumn, and 339 ha (95% CI [205, 566]) in winter; however, we did not detect a significant difference between seasonal estimates using Tukey-adjusted contrasts (*P* > 0.05). Predicted range overlap (0–1) was 0.48 (95% CI [0.33, 0.62]) in spring, 0.40 (95% CI [0.31, 0.50]) in summer, 0.44 (95% CI [0.33, 0.54]) in autumn, and 0.37 (95% CI [0.27, 0.46]) in winter. Range overlap (transformed) was significantly greater during summer compared to winter (estimate = 0.18, SE = 0.07, t = 2.62, *P* = 0.04) but did not differ between other seasons (*P* > 0.05). Range overlap was significantly reduced for one day following capture (Fig 5). The average long-term range overlap model-predicted marginal mean was 0.40 (95% CI [0.31, 0.50]) (Fig 5).

## Discussion

Overall, drop net and helicopter capture methods were safe for white-tailed deer with only 3 direct capture moralities (1.6% of 190 capture events) and 10 additional mortalities (5.3% of 190 capture events) within the first 31 days after capture. Our study was designed to assess potential effects of capture on white-tailed deer space use, movement, and survival, but human safety is of utmost importance, so wildlife professionals should be mindful of the inherent risks to human safety associated with helicopter operations, particularly when using multiple helicopters. Capture methods had minimal effect on deer movement and space use with deer typically returning to normal behavior within 1–2 days, as evidenced by displacement patterns of female and male deer captured by single helicopter and tandem helicopters and range overlap. In rare cases, deer exhibited atypical behavior, including suppressed movement lasting up to 9 days following capture by a single helicopter or 5 days following capture by tandem helicopters. High post-capture survival (*S* = 0.93 ± 0.02 SE across sexes) and minimal behavioral impacts, except in the short-term (typically 1–2 days but as long as 9 days), provide greater confidence for behavioral and movement studies that deer were not adversely impacted by capture. We evaluated multiple space use and movement metrics made possible by leveraging subhourly location data, rather than relying on a single measure, typically movement alone. However, space-use metrics contributed little additional information beyond what was captured by movement and displacement. We therefore recommend that researchers prioritize distance traveled (movement rates) and displacement when assessing capture effects on large mammal behavior, particularly when developing protocols to censor atypical post-capture location data.

While human safety must always be the foremost concern during capture operations, animal welfare remains an important consideration when selecting the most appropriate capture method to meet research objectives [83]. Survival was high (S ≥ 0.90) for all capture methods, with little variation among them (S = 0.90 [drop nets]–0.94 [helicopter methods]). Capture-induced mortality rates were at or near the suggested threshold for safe capture (≤2%) [83], with estimates of 3.2% for drop net, 1.5% for single helicopter, and 1.1% for tandem helicopter. Comparable survival rates among these capture methods have also been reported in previous studies [14,22]. For example, Kock et al. [22] reported 1% accidental mortality of bighorn sheep (*Ovis canadensis* spp.) using drop nets and 2% accidental mortality from helicopter net-gunning; they also reported that capture myopathy was 2% and 0% using drop net and helicopter net-gun techniques, respectively. Likewise, White and Bartmann [14] reported that mule deer (*Odocoileus hemionus*) fawn survival was 90% and 95% at 2-weeks and 83% and 89% at 4-weeks following capture by drop net and helicopter net-gun techniques, respectively. Our findings, and those of others, show that drop net and helicopter net-gun capture methods are effective and pose minimal risk to animal welfare when capturing large mammals (S ≥ 0.90; [14,16–18,20,36,37,49,59,61,84,85]).

Deer movement and space use typically returned to long-term means within 1–2 days, but as long as 5 days or 9 days in one case each, and these prolonged periods only applied to distance traveled by deer following capture by single or tandem helicopters. Short-term behavioral responses have been documented across large mammals following capture by helicopter net-gun [24,35–37] and other capture techniques [31,38,60,74,86–89]. In contrast, Dechen Quinn et al. [33] found that white-tailed deer movement was reduced for up to 14 days following capture by clover traps, rocket nets, Stephenson box traps, and dart guns; all of which used chemical sedatives to immobilize deer during processing. Herein, we did not use chemical immobilization, so the high survival and lack of behavioral responses may partially be attributed to not using chemical immobilization. Regardless, it is common practice, and recommended, to capture large mammals without the use of chemical immobilization when possible because chemical immobilization can have deleterious effects [22,29,90,91].

Capture methods affected space use and movement metrics differently, or not at all. Typically, deer captured with drop nets showed little to no change in movement and space use, while those captured by helicopter net-gun capture exhibited short-term changes in movement metrics. This difference in behavioral response following capture by drop net and net-gun helicopter capture methods may be, at least partially, explained by basic deer ecology. White-tailed deer demonstrate strong fidelity to their home ranges and core areas of use [92,93], which are relatively small and stable [93]. The bait (shelled corn) used in this study likely attracted deer within their home ranges, so, when released from drop nets, deer were likely close to or already within established ranges, which minimized the effect of capture on deer spatial behavior. In contrast, deer captured by helicopters had the greatest response because they were transported <0.5 km (tandem helicopters) or 0.5–3.1 km (single helicopters), which likely was outside of or on the periphery of established ranges, necessitating greater movement to return to these areas. We found support for this explanation in our long-term displacement estimates for female deer captured by single helicopters, which settled, on average, 1.6–1.7 times farther from release sites compared to those captured by drop nets and tandem helicopters, respectively. Similarly, Northrup et al. [24] reported that deer captured by helicopter and transported 2–5 km away returned to their home ranges within 14 hours but occasionally took up to 75 days.

Researchers are often burdened with time and economic constraints, which may influence what capture method will best meet research objectives [14,15]. Drop nets can be the least economical and most time intensive method among the three [14,15], depending on factors relating to the density of the target species, capture effort, and landscape composition. In a companion study, Beaver et al. [15] found that drop net captures were nearly 4 times and 3 times more costly than single and tandem helicopter captures, respectively, with most of the cost (~80%) being attributed to personnel expenses. Similarly, White and Bartmann [14] reported a 66% increase in cost per deer fawn captured by drop nets compared to net-guns deployed from a helicopter because of personnel costs. However, net-gun captures involve risks to human safety and may be less effective or entirely ineffective in heavily forested systems [14,25,27], decreasing capture efficiency and reducing the net economic benefit. Likewise, drop nets may be a preferred option near human development where use of helicopters may be logistically impossible [17,94]. Thus, researchers should first consider the landscape and behavior of the species before deciding on the best capture method.

Previous studies have recommended that researchers conduct investigations into location datasets prior to formal analysis to identify and subsequently censor data biased by capture [24,33,37]. We extend this recommendation by suggesting that researchers incorporate distance traveled (movement), displacement from the capture site, and, when feasible, space use metrics to better evaluate capture effects on large mammals, recognizing that animal behavior is complex and best assessed holistically. In the context of reintroductions, Berger-TAL and Saltz [95] recommended that researchers and managers evaluate release site fidelity (i.e., displacement), recurring locations (i.e., range overlap), proximity to other individuals, and individual variation in movement behavior (i.e., distance traveled) to identify when translocated animals settle into their new environment. Similarly, Baumgardt et al. [37] evaluated the daily distance traveled and displacement of nilgai (*Boselaphus tragocamelus*) in southern Texas to isolate the effect of capture. We found that evaluating distance traveled and displacement of white-tailed deer provided unique insights into the spatial dynamics. Although space use metrics were not particularly informative in our study, we recognize their inclusion may further expand these insights. If an investigation into the dataset cannot be conducted, we suggest that researchers censor the first day or two of location data following release, with a more conservative approach extending up to 9 days [24,31,36–38,60,74,86–89].

We balanced multiple competing objectives when determining what dates to capture deer and the length of collar deployment, which resulted in an uneven sample distribution, particularly females captured by drop nets, a limited number of recapture events, and typically <1 year of location data per collar deployment. Previous studies have compared location data collected from captured animals before and after a subsequent capture [24,37,60,86] or location data from the days following capture to location data collected on the same Julian days of which animals had resumed normal activity [33]. However, both methods were unavailable to us due to a limited number of recaptures and collar deployment length. A common approach to evaluate the effect of capture is to compare metrics after capture to some predetermined time frame that represents “normal activity” [34,38,60,74]. We chose a similar approach; however, instead of using the literature to predict when “normal activity” likely occurred [34], we created a long-term mean for each individual deer by averaging the movement and space use metrics for each deer across the entire 30-day period [74], thereby controlling for individual-specific differences [37]. We suggest that our methodological approach may be useful for other before-after study designs when assessing the impacts of capture, relocation, or similar animal disturbing activities.

## Conclusions

The 3 capture methods had little to no effect on survival and post-capture behavior of white-tailed deer. Therefore, the choice of capture method may be more strongly influenced by landscape characteristics, vegetation cover, and risks to human safety, with helicopter-based methods being generally better suited for open terrain, while drop nets may be more appropriate in densely wooded areas or near human development [18,23–26]. Deer movement and displacement tended to be more affected by helicopter capture methods, which was likely influenced by the distance the animal was moved outside their range [24]. Drop nets may be advantageous to helicopter net-gun capture methods when attempting to minimize the behavioral response of deer to capture. However, drop nets can be the least economical and most time intensive method among the three [14,15], depending on landscape conditions. We recommend researchers conduct investigations into location datasets prior to formal analysis to identify and subsequently censor data affected by capture, which could bias certain analyses. Researchers should, at minimum, incorporate distance traveled (movement) and displacement to fully understand the effects of capture on animal behavior thus minimizing anomalies, albeit temporary, and their effects on subsequent analyses. However, if an investigation into the dataset cannot be conducted, we recommend that researchers censor the first day or two of location data following release, with a more conservative approach extending up to 9 days, to reduce the possibility of including data biased by capture [34,37].

## Supporting information

S1 Code Survival data and analysis. R script and tutorial used to perform survival analyses and create figures included in the manuscript. The data is that of female and male white-tailed deer (*Odocoileus virginianus*) on Joint Base San Antonio-Camp Bullis, Texas, USA, 2011–2015.
(ZIP)

S2 Code Location data and movement and space use analyses. R script and tutorial used to conduct data processing, perform movement and space use analyses, and create figures included in the manuscript and reported within the supplementary files. The location data is that of female and male white-tailed deer (*Odocoileus virginianus*) on Joint Base San Antonio-Camp Bullis, Texas, USA, 2011–2015.
(ZIP)
