# Supplementary material for: Effects of drop net and helicopter net-gun capture on movement, space use, and survival of white-tailed deer
Source: PLoS One. 2026 Jan 6;21(1):e0340491. doi: 10.1371/journal.pone.0340491 (PMC12798857; doi:10.1371/journal.pone.0340491)
Supplement: S1 Code — R script and tutorial used to perform survival analyses and create figures included in the manuscript. The data is that of female and male white-tailed deer (Odocoileus virginianus) on Joint Base San Antonio-Camp Bullis, Texas, USA, 2011–2015. (ZIP) [file pone.0340491.s001.zip › S1 Code/Data/Read Me.docx]

The data file, ‘Morts.csv’, has 9 column headings, which are described below:

**AnimalID**: This is the unique animal identifier, which is individual specific.

**KC_CollEv**: This is the individual capture identifier. One individual can have multiple capture identifiers if the animal was captured more than once.

**Sex**: This identifies the deer as male or female.

**time**: This represents how many days the deer survived following release. If the value is 30, then the animal survived beyond the study period. A value of 0 represents the deer died on the day of capture.

**Censor**: Zeros represent deer that were alive at the end of the study; ones represent deer that died within the 31-day study period (day of capture + 30 days).

**CaptureMethod**: This is what method was used to capture the animal.

**RecapNumber**: This represents whether the animal was captured for the first time (NoRecap) or recaptured (Recap).

**Season**: This represents the season of capture (spring, summer, autumn, winter).

**Season2**: This represents the reclassified seasons: cool (spring, autumn, winter) and warm (summer).
