# Supplementary material for: Effects of drop net and helicopter net-gun capture on movement, space use, and survival of white-tailed deer
Source: PLoS One. 2026 Jan 6;21(1):e0340491. doi: 10.1371/journal.pone.0340491 (PMC12798857; doi:10.1371/journal.pone.0340491)
Supplement: S2 Code — R script and tutorial used to conduct data processing, perform movement and space use analyses, and create figures included in the manuscript and reported within the supplementary files. The location data is that of female and male white-tailed deer (Odocoileus virginianus) on Joint Base San Antonio-Camp Bullis, Texas, USA, 2011–2015. (ZIP) [file pone.0340491.s002.zip › S2 Code/Data/Read Me.docx]

The data file, ‘PostCapDeer.csv’, has 19 column headings, which are described below:

**AnimalID**: This is the unique animal identifier, which is individual specific.

**KC_CollEv**: This is the individual capture identifier. One individual can have multiple capture identifiers if the animal was captured more than once.

**Sex**: This identifies the deer as male or female.

**UTM_X:** These are the longitude coordinates in UTM.

**UTM_Y:** These are the latitude coordinates in UTM.

**TimeStamp_LMT:** This is the timestamp in local mean time.

**Date_LMT:** This is the date in local mean time.

**Sunrise_LMT:** This is the sunrise time in local mean time.

**Sunset_LMT:** This is the sunset time in local mean time.

**CaptureMethod**: This is what method was used to capture the animal.

**Recap**: This represents whether the animal was captured for the first time (NoRecap) or recaptured (Recap).

**StepLength**: This is the Euclidean or straight-line distance between sequential GPS locations.

**TimeStamp_UTC**: This is the timestamp in UTC.

**Min_BN_Dec:** This is the number of minutes that elapsed between sequential GPS locations.

**DaysFromCapture:** This is the day following capture.

**DaysInStudy:** This is how many days the deer was in the study. All deer within this dataset were in the study for the entire 30-day period.

**Season:** This is the capture season (spring, summer, autumn, or winter).
